# Thyroid Cancer in the Pediatric Population

**DOI:** 10.3390/genes10090723

**Published:** 2019-09-18

**Authors:** Vera A. Paulson, Erin R. Rudzinski, Douglas S. Hawkins

**Affiliations:** 1 Department of Laboratory Medicine, University of Washington Medical Center, 1959 NE Pacific St, Box 357110, Seattle, WA 98105, USA; 2 Department of Laboratories, Seattle Children’s Hospital, OC.8.720; 4800 Sandpoint Way NE, Seattle, WA 98105, USA; erin.rudzinski@seattlechildrens.org; 3 University of Washington Medical Center, Fred Hutchinson Cancer Research Center and Cancer and Blood Disorders Center, Seattle Children’s Hospital, MB.8.501, Seattle, WA 98105, USA; doug.hawkins@seattlechildrens.org

**Keywords:** pediatric thyroid cancer, molecular testing, gene rearrangements, targeted cancer therapy

## Abstract

Thyroid cancer is rare in the pediatric population, but thyroid carcinomas occurring in children carry a unique set of clinical, pathologic, and molecular characteristics. In comparison to adults, children more often present with aggressive, advanced stage disease. This is at least in part due to the underlying biologic and molecular differences between pediatric and adult thyroid cancer. Specifically, papillary thyroid carcinoma (which accounts for approximately 90% of pediatric thyroid cancer) has a high rate of gene fusions which influence the histologic subtypes encountered in pediatric thyroid tumors, are associated with more extensive extrathyroidal disease, and offer unique options for targeted medical therapies. Differences are also seen in pediatric follicular thyroid cancer, although there are few studies of non-papillary pediatric thyroid tumors published in the literature due to their rarity, and in medullary carcinoma, which is most frequently diagnosed in the pediatric population in the setting of prophylactic thyroidectomies for known multiple endocrine neoplasia syndromes. The overall shift in the spectrum of histotypes and underlying molecular alterations common in pediatric thyroid cancer is important to recognize as it may directly influence diagnostic test selection and therapeutic recommendations.

## 1. Introduction

Thyroid cancer is the leading cause of pediatric endocrine cancer, accounting for over 6% of all pediatric cancers from 2012 to 2016 [1]. This figure reflects the rising incidence of pediatric thyroid carcinoma over the last four decades [1]. Between 1975 and 1995, the annual percent change (APC) of pediatric thyroid carcinoma was +0.8%/year; with an accelerated +4.6%/year APC from 1996 to 2016 [1]. The majority of this increase is comprised of the most common subtype of thyroid cancer—papillary thyroid carcinoma (PTC). However, increases are also reported for follicular thyroid carcinoma (FTC) [2]. The contribution of medullary (MTC), anaplastic (ATC), and poorly differentiated (PDTC) thyroid carcinomas is minor given their rarity in the pediatric population [1,3,4,5]. Several studies suggest that the increasing incidence of papillary and follicular thyroid carcinoma is only partially accounted for by surveillance and enhanced detection, as there is also an increase in detection of large tumors and advanced-stage disease [3,6]. However, Chen et al. suggest that the increased rate of thyroid carcinoma may be the result of increased detection secondary to a “reservoir of clinically silent cancers”, increased use of medical imaging, and increased imaging sensitivity [7].

Historically, recommendations for the evaluation and management of thyroid cancer in the pediatric population have been extrapolated from adult guidelines. However, compared to adults, thyroid cancers in the pediatric population differ in clinical presentation, pathophysiology, and long-term outcomes [8]. The American Thyroid Association (ATA) has therefore published guidelines specifically addressing the management of children with thyroid nodules and differentiated thyroid cancer (DTC) in 2015 [8]. Herein we review the clinicopathologic presentation and treatment of pediatric thyroid carcinoma, highlighting key morphomolecular findings and their impact on clinical management.

## 2. Background and Epidemiology

Per the Surveillance, Epidemiology, and End Results program, 1.8% of the thyroid cancers diagnosed in the US are in children and adolescents [1]. Rarely neonatal thyroid carcinoma has been described [9], but most cases of pediatric thyroid carcinoma occur in the second decade [1]. Increased age also results in an increasingly disproportionate number of females affected compared to their male peers, with nearly a 6:1 ratio by 15–19 years of age [1]. The vast majority of these thyroid cancers are PTC (80–90%) followed by FTC (~10%), MTC (3–5%), and rarely ATC and PDTC [4,5,8,10,11]. Reported risk factors in the development of follicular-cell derived thyroid cancer in children include thyroid disease (e.g., autoimmune thyroid disorders) [8,12], nutritional (i.e., iodine) deficiency [13], and prior radiation exposure [8,14,15,16]. The frequency of malignancy in pediatric patients with Hashimoto’s thyroiditis has been reported to range from 0.67% to ~3%, which is above the background risk of ~0.02% in the general pediatric population (based on an incidence rate of 1.14/100,000 per year over the course of 19 years) [1,3,12,17,18,19]. Proposed mechanisms for the association between Hashimoto’s thyroiditis and PTC include over-production of thyroid stimulating hormone (TSH) or chronic inflammation resulting in proliferation, angiogenesis, and/or reduced apoptosis [12]. Data in pediatric Graves’ disease is more limited with respect to malignancy risk, with reports of follicular-cell derived thyroid cancer ranging from <1% to 22% of patients undergoing definitive surgical therapy; this number further decreases if non-surgically treated Graves patients are included in the denominator [20,21,22]. The link between iodine deficiency and FTC has also been more extensively studied in adults; however, of the few studies published in the pediatric population, several have suggested that iodine intake may be protective in the setting of radiation exposure and the development of PTC [13,23,24,25]. Radiation exposure, either environmental, as part of diagnostic tests, or following radiotherapy for a prior malignancy (most commonly Hodgkin lymphoma or central nervous system tumors) also increases the risk of DTC [8,14,15,16]. Indeed, thyroid cancer is one of the most common secondary malignancies among survivors of childhood cancer [8,14,15,16]. 

Rarely genetic predisposition syndromes including *APC*-associated polyposis, *DICER1* syndrome, Carney Complex, *PTEN* hamartoma/Cowden syndrome, and Werner syndrome are associated with DTCs, accounting for approximately 5% of cases [6,8,26]. Cases of PTC and FTC have also been reported in association with Li–Fraumeni, Peutz–Jeghers, familial paragangliomas, McCune–Albright, and Beckwith–Wiedemann [8]. By comparison, the vast majority of MTC in the pediatric population are hereditary, the result of germline *RET* mutations resulting in multiple endocrine neoplasia (MEN) type 2A syndrome (90–95% of childhood MTC), MEN type 2B, or familial medullary thyroid carcinoma (FMTC) [6,27,28]. 

## 3. Clinical Presentation

A thyroid nodule in the pediatric patient typically manifests as an asymptomatic neck mass, with or without cervical lymphadenopathy, and may be accompanied by trouble breathing and/or hyperthyroidism. The identification of a nodule is clinically important due its increased malignant potential in the pediatric population. Nodules in the adult population are relatively common, occurring in 19–68% of the population, but are rarely malignant (5–10% of all thyroid nodules in adults, per most series) [8,29,30,31]. The opposite is true in children and adolescents. Approximately 1–3% of children harbor thyroid nodules, and more than a quarter (22–26%) of thyroid nodules occurring in pediatric patients are malignant [6,8,30,31].

A large hard nodule, especially one adherent to adjacent tissue, is concerning for cancer [6]. Equally concerning are nodules determined to have suspicious ultrasonographic features by ATA guidelines, including irregular margins, marked hypoechogenicity, and microcalcifications, or nodules accompanied by pathologic adenopathy [30,32]. Solid and predominantly solid nodules are also more likely to be malignant compared to cystic nodules [32]. It is worth noting that the diffuse sclerosing variant of PTC may present as diffusely infiltrating disease resulting not in a single nodule but in diffuse enlargement of a lobe or the entire gland [33,34]. Diffuse thyroid enlargement, especially if accompanied by cervical lymphadenopathy, must therefore prompt imaging for the microcalcifications that are invariably associated with this subtype [33,35]. Whether the ATA criteria for malignancy risk by ultrasound (US) features directly apply to pediatric thyroid lesions is a subject of continued investigation. Creo et al. demonstrated that revised criteria for risk of malignancy based on institutional report of local radiologists overall impression was more specific for a diagnosis of thyroid cancer in children as compared to ATA guidelines [31,36] but there was diminishing accuracy of these guidelines for predicting malignancy in younger patients [36].

Per recent pediatric management guidelines, the identification of a solitary or suspicious thyroid nodule on imaging (with features described above) or physical examination should prompt utilization of a diagnostic algorithm that includes evaluation of TSH (and nuclear thyroid scintigraphy in the setting of TSH suppression) to determine whether the nodule should undergo US-guided fine needle aspiration (FNA) (if hypofunctioning) or surgical resection (if hyperfunctioning) [8]. In the pediatric population, hyperfunctioning thyroid nodules by scintigraphy appear to carry a risk of malignancy of <10–15% [37,38,39], though some reports suggest a higher risk of malignancy than in adults (up to 30%) [40]. The Bethesda classification is used to predict the risk of malignancy based FNA findings, as discussed below [30,41].

## 4. Pathologic Evaluation

### 4.1. Fine Needle Aspiration

FNA has long been a mainstay in the diagnostic work-up of adult thyroid nodules, and recent literature supports its efficacy in the pediatric population, with 99% accuracy and sensitivity and specificity of 94% and 100% respectively, per one recent study [41]. The Bethesda system classifies thyroid FNAs into six categories, each category linked to a malignancy risk [30,41]. Although there is significant variability between studies, the risk of malignancy in non-diagnostic (Bethesda I), benign (Bethesda II), and suspicious/malignant (Bethesda V/VI) mirror adult risk assessment at 0% (range 0–10%), 5–8% (range 0–16%), and 100% respectively (Bethesda V range: 40–100%, Bethesda VI range: 100%). [42]. Several studies suggest that Bethesda III (atypia of uncertain significance/follicular lesion of uncertain significance; AUS/FLUS) and Bethesda IV (follicular neoplasm/suspicious for follicular neoplasm; FN/SFN) categories account for up to 40% (range: 13–43%) of all pediatric FNA diagnoses [42]. Further, these indeterminate aspirate diagnoses are associated with increased risk of malignancy in children versus adults (28% in children vs. 6–30% in adults for AUS/FLUS and 58% in children vs. 10–40% in adults for FN/SFN lesions) [42]. However, more recent studies indicate lower percentages of indeterminate FNA groups than previously reported (13–18% for indeterminate diagnoses combined) and a lower risk of malignancy after histologic resection within these groups (11–20% for AUS/FLUS; 25–28% for FN/SFN) [42].

### 4.2. Surgical Resection

Although diagnostic criteria for PTC are the same regardless of patient age, differences in histotypes and overall stage occur between adult and pediatric populations. Children tend to present with nodal disease and evidence of local or distant metastases [8]. They also have a higher risk of recurrence than adults with thyroid carcinoma. Small studies suggested that younger age, male sex, multifocality, and tumor size greater than 2 cm predicted extensive disease at diagnosis [43]. However, Balachandar et al. suggest that the primary predictor of extent of disease is the presence of extensive extrathyroidal extension, regardless of any other clinical or pathologic features [43]. High risk histologic subtypes of PTC are reported to occur in 15–37% of pediatric PTC, including 7–13% tall-cell variant, 7–16% diffuse sclerosing variant, 1–4% solid/trabecular variant and 2–6% poorly differentiated carcinoma. While some studies suggest the solid/trabecular variant may have an increased recurrence risk in children, most studies indicate that these “high-risk” histologic subtypes do not carry a worse event free survival in children [44,45]. This is in contrast to the increased risk of recurrence associated with these histologic subtypes reported in the adult population [43]. Low risk subtypes (classic and follicular variants of PTC) remain the most commonly encountered subtypes, while the newly described noninvasive follicular thyroid neoplasm with papillary-like nuclear features (NIFTP) accounts for nearly 4.5% of pediatric PTC [46]. 

Follicular carcinoma in children is usually minimally invasive (90% of all FTC cases), rather than widely invasive [47,48]. Thus, as opposed to PTC, children with FTC have less aggressive disease than adults.

Medullary carcinoma is infrequently encountered in the pediatric population; however, prophylactic thyroidectomy is routinely performed for children with MEN2A, with surgical timing based on risk associated with specific mutations [28]. In this context, C-cell hyperplasia (CCH) is the most common finding [49]. Neoplastic CCH is considered distinct from physiologic CCH, and represents the precursor lesion of MTC. Cytologic atypia and integrity of the basement membrane are helpful in distinguishing the neoplastic pattern of CCH from either physiologic CCH or medullary microcarcinoma, respectively [50,51,52,53]. 

## 5. Molecular Diagnostic Testing

Molecular diagnostic testing has several emerging roles in the management of children with thyroid lesions. First, although data is scant in children, early studies suggest that indeterminate FNA diagnoses (AUS/FLUS and FN/SFN) may be further stratified by molecular findings [54]. Monaco et al. determined the presence of classic *RAS* and *BRAF* mutations as well as *RET-PTC* and *PAX8-PPARG* fusions in pediatric nodules that had undergone FNA [54]. They reported that all mutation positive FNAs were PTC on resection [54]. In general, however, given the difficulties of performing repeat FNA procedures in young children who may require sedation and the current ATA guidelines which recommend surgery (lobectomy plus isthmusectomy) for atypical thyroid nodules, molecular testing of FNA material is often deferred from routine practice [30]. More studies are needed to define the role of molecular testing in indeterminate thyroid nodules in the pediatric population. 

The spectrum of genetic alterations found in pediatric thyroid carcinoma is shifted in comparison to what is found in the adult population. As most pediatric studies on the molecular landscape of thyroid carcinoma have been driven by findings reported in the adult population, the overall alterations described in pediatric and adult thyroid cancer populations are similar. Oncogenic gene fusions occur with increased frequency in pediatric thyroid carcinoma, with 50–60% of pediatric thyroid cancers harboring a fusion compared to 15% in the adult population (see Table 1). Correspondingly, point mutations occur with decreased frequency, with 30% of pediatric thyroid cancers demonstrating point mutations compared to nearly 70% of adult thyroid cancers [14,26]. These differences drive both potential diagnostic test selection (e.g., ensuring inclusion of fusion genes in molecular analysis of indeterminate aspirate samples) and therapeutic considerations.

### 5.1. PTC

Given its predominance in the pediatric population, the most extensively studied pediatric thyroid cancer, in relation to molecular drivers, is PTC. The molecular results of approximately 2000 “pediatric” PTCs have been reported in the literature (see Table 1); however, their characterization has been limited to small cohorts assessed for select alterations or in more recent years small multi-gene panels for the detection of point mutations or specific gene rearrangements (e.g., thyroseq) [26,51,54,55,56,57,58,59,60,61,62,63,64,65,66,67,68,69,70,71,73,74,75,76,77,78,79,80,81,82,83,84,85,86,87,88,89,90,91,92,93,94,95,96,97,98,99,100,101,102,103,104]. The combination of these small cohorts with varying demographics (namely the age range included as “pediatric”), geographical and environmental factors, and method of assessment often results in a wide range of frequencies for the observed genetic alterations.

*RET* fusions are reported to be the most common observed alteration, and by our review appear to occur in approximately 25–30% of sporadic pediatric PTC (range 14–55%, Table 1), a figure that further increases to nearly 45% (range: 14–87%, Table 1) in patients exposed to radiation. While new *RET* fusion partners are continuously identified and reported, the most common are *PTC1* and *PTC3*, though there is some variability in frequency due to detection methods and expression heterogeneity [26]. *RET* fusions are associated with classic, solid, and diffuse sclerosing PTC subtypes as well as increased extrathyroidal extension, spread to locoregional lymph nodes, and distant (pulmonary) metastases [26,103]. It should be noted that *RET-PTC* fusions have also been reported in benign thyroid samples, more commonly in those with than without prior radiation exposure [72,105,106]. 

Although less common than *RET*, other oncogenic fusions occur with increased frequency in sporadic pediatric PTC. Reported fusions include *NTRK*(*1*, *2* and *3*), *BRAF*, and *ALK*. Although not comprehensively surveyed, these fusions have been identified in approximately 10% (range: 0–26%), 10% (range: 0–18%), and 5% (range: 0–21%) of pediatric PTC, respectively (see Table 1).

*BRAF* point mutations are reported to be the second most common single oncogenic driver in sporadic pediatric PTC, though our comprehensive review suggests *BRAF* mutations may occur as frequently as *RET-PTC* fusions (25–30% of lesions, range: 0–63%, Table 1). While more than 40 mutations have been identified in *BRAF* to date, >95% are V600E, though this may be skewed by methodology as many of these studies only assessed this single mutation. BRAFV600E mutations are associated with classic PTC histology and correlate with higher age at diagnosis; they do not appear to portend a more aggressive clinical course [96]. 

*RAS* mutations (specifically HRAS Q61R and NRAS Q61K) are present in <5% (range: 0–18%, Table 1) of sporadic pediatric PTC, and are more frequent in benign thyroid nodules (15–40%) [57,99]. *RAS* mutations were more commonly associated with the follicular variant of PTC, and undoubtedly some would be reclassified as NIFTP in light of current practices [26,87]. The clinical implications and impact of these *RAS* mutations are therefore difficult to discern given their identification in both benign and varied malignant lesions. 

*PAX8-PPARg* fusions have also rarely been reported in pediatric PTC (<5%, range 0–9%, Table 1), predominantly the follicular variant though two solid variant PTCs were reported in Ricarte-Filho [26,80]. More comprehensive molecular panels have rarely identified mutations in *DICER1* (up to 10% in one study), *TP53*, *TERT*, *PIK3CA*, and *PTEN* [57,80,86,100]. 

### 5.2. FTC and PDTC

Little is known about the drivers of pediatric FTC, with less than 100 cases scattered predominantly throughout reported cohorts of DTC (Table 1) [54,57,65,66,68,74,76,86,87,88,89,90,99,101]. A minority appear to harbor activating *RAS* point mutations (10–15%, range: 0–20%) and *PAX8-PPARg* fusions (<5%, range 0–20%), neither of which is unique to FTC (see Table 1). Cohort size (0–41) has limited meaningful analysis of the association of clinicopathologic and molecular findings. Even less is known about pediatric PDTC, with only three cases included in the analyzed cohorts [74,85,90]. 

### 5.3. MTC

Unlike follicular derived tumors, and MTCs in the adult population, over 95% of pediatric MTCs are hereditary, secondary to activating *RET* mutations [6,27]. Moreover, of the apparently sporadic cases of MTC another 6–10% are de novo germline *RET* mutations [28]. Gain of function *RET* mutations occur in two different ways. Either the mutation occurs in one of the six cysteine residues within the extracellular domains (Cys609, 611, 618, 620, 630, and 634) and promotes dimerization and ligand-independent activation, or the mutation occurs in the tyrosine kinase domain and confers ligand independent catalytic activity in monomeric form [28]. 

### 5.4. Molecular Summary

Compared to the landmark TCGA study in 2014, which reduced unknown oncogenic drivers in adult PTC from 25% to 3.5% through exome and whole genome DNA sequencing, RNA sequencing, miRNA sequencing, single nucleotide polymorphism (SNP) arrays, DNA methylation arrays, and reverse phase protein arrays of nearly 500 cases of PTCs (and of which only nine were pediatric), the pediatric PTC studies are quite limited [107]. Moreover, given the small pediatric cohorts, variety of methodologies used, and wide range of the molecular findings, it is difficult to know the true frequency of these alterations in the pediatric population and whether we have truly defined the molecular characteristics of this entity. If PTC is understudied, FTC and PDTC studies are virtually non-existent in pediatrics. Additional efforts with broader panels and larger cohorts are needed if we are to better define the genomic landscape of pediatric thyroid cancer and whether there are associations between histology and/or outcome. While molecular diagnostic testing is of uncertain utility in the setting of indeterminate FNAs and borderline histologic diagnoses; its role in treatment in certain settings is paramount. 

## 6. Treatment

Total thyroidectomy is the treatment of choice for pediatric PTC and FTC due to the increased incidence of bilateral (30%) and multifocal (65%) disease [8], though a near-total thyroidectomy, in which a small amount of thyroid tissue near the recurrent laryngeal nerve or superior parathyroid glands may be spared to decrease the possibility of damage to those structures, is also an option. While data in the adult thyroid cancer population suggests there may be a role for less extensive surgery in certain clinical contexts, such an approach is less common in the pediatric population due to the increased risk of local recurrence in patients treated by lobectomy [108]. In the presence of central or lateral neck metastases, thyroidectomy should be accompanied by a central or lateral neck dissection. Neck dissection can also be considered prophylactically based on tumor size and focality though its use must be weighed against possible complications. The ATA task force further recommends adjuvant radioactive iodine (RAI) for unresectable iodine-avid persistent locoregional disease (due to invasion of vital structures) and/or distant metastases, which may be further guided by post-operative thyroglobulin levels [7,8]. The pediatric recommendations regarding indications for RAI differ from those published by the National Comprehensive Cancer Network for adults with PTC, which cite a variety of clinical features including primary tumor size >2–4 cm, gross extrathyroidal extension, and extensive or bulky regional nodal involvement as indications for adjuvant RAI [109]. In contrast, the guidelines for children with DTC recommend using post-thyroidectomy TSH-stimulated thyroglobulin levels (in addition to TSH-stimulated ^123^I imaging and the presence of distant metastases) to determine who should receive adjuvant RAI [8]. However, this algorithm assumes the absence of an anti-thyroglobulin antibody, with the recommendation to use only extensive local invasion or distant metastases as indications for RAI for patients with an anti-thyroglobulin antibody and potentially delay RAI to allow the anti-thyroglobulin antibody to clear prior to committing to RAI. There is no consensus on the preferred dose adjustment method to calculate a ^131^I for a child, with body weight and body surface area methods described. Whole body ^131^I dosimetry can also be used in patients with extensive metastases or who require repeat RAI to limit organ doses. For additional information regarding patient preparation for and administration of RAI, the authors recommend an excellent review of RAI by Parisi et al. [110]. 

Despite more advanced disease at presentation, 40–90% extensive regional node involvement and 7–30% distant lung metastasis, PTC has an excellent prognosis with greater than >98% survival, by most studies [7,46,111]. For patients with DTC who recur, RAI is usually effective. For patients with iodine non-avid or RAI-refractory disease or residual/metastatic MTC, molecularly targeted kinase inhibitors (TKI) may provide a medical therapeutic option.

Most of our knowledge of TKIs and their efficacy comes from the adult population extended to pediatric patients through ongoing clinical trials as well as case reports and small case series in the literature. To date, several kinase inhibitors (many of which are non-specific) have been approved for the treatment of thyroid cancer. Vandetanib and cabozantinib have received FDA approval for the treatment of MTC, while lenvatinib and sorafenib (regardless of mutational status), combination dabrafenib/trametinib (in the setting of anaplastic thyroid carcinoma and BRAFV600E), and larotrectinib (in the setting of *NTRK* fusions in any tumor type including thyroid) have been approved for DTC. Studies evaluating the efficacy of additional treatment options, including inhibitors of *RET* alterations and *ALK* fusions, are ongoing in both adult and pediatric patients with thyroid cancer. Table 2 provides a summary of the therapies (either with a specific approved indication or investigated in a recent clinical trial) according to their targeted molecular alterations and/or tumor type.

Several of the therapies mentioned above nonspecifically target RET, including vandetanib, cabozantinib, lenvatinib, and sorafenib. While their utility has been proven in *RET* mutation-positive thyroid cancers and thyroid cancer with unknown mutational status, their efficacy (along with that of ponatinib, sunitinib, LOXO-292, BLU-667) in *RET* fusion-positive thyroid cancer has not yet been fully established. Vandetanib demonstrated both a marked objective response (44% vs. 1%) and progression free survival (hazard ratio, 0.35) compared to placebo for the treatment of symptomatic or progressive unresectable, locally advanced, or metastatic medullary thyroid cancer in adult patients [112,113]. Children with locally advanced or metastatic MTC receiving vandetanib also showed a marked response with a confirmed objective partial response rate of 47%, as well as a median progression free survival of 6.7 years and a 5-year overall survival of 88.2% [114,115]. Advanced RAI-refractory DTC in adults has also shown promising results in a recent phase II trial of vandetanib (hazard ratio 0.63) though preliminary results of a phase 3 trial merely showed a trend favoring vandetanib and were not statistically significant [116]. Cabozantinib appears equally efficacious in adult patients with unresectable and/or locally advanced MTC with a 29% partial response and a statistically significant longer progression free survival (hazard ratio 0.28) [116,117]. To date only five pediatric patients with MTC have received cabozantinib as part of a larger study of dosage and pharmacodynamics; two demonstrated confirmed partial responses and two were observed to have stable disease [118]. Cabozantinib is also being investigated in the context of adult differentiated thyroid cancer, with promising phase I and II trial results, with a partial response of 45% and 40% respectively [116]. In the setting of adult late-stage metastatic and progressive radioactive iodine-refractory differentiated thyroid carcinoma, sorafenib and lenvatinib have both shown improved progression free survival compared to placebo, 10.8 months vs. 5.8 months (hazard ratio 0.59) and 18.3 months vs. 3.6 months (hazard ratio 0.21) respectively [116,119,120]. In the pediatric population, response to sorafenib has been reported both in progressive RAI refractory PTC, PTC with diffuse metastatic disease not amenable to upfront RAI, and as gap therapy in a patient who could not receive RAI in a timely fashion (one case each) [121,122,123], while stable disease has been reported in three pediatric patients with extensive bilateral metastatic pulmonary disease treated with lenvatinib (including one previously treated with sorafenib) [124]. Clinical trials evaluating the efficacy of lenvatinib in the treatment of children with refractory or relapsed solid malignancies (including thyroid cancer) are ongoing. More potent (and specific) RET inhibitors may be on the horizon according to interim clinical data reported at the 2018 American Thyroid Association (ATA) and American Association for Cancer Research (AACR) annual meetings. Both LOXO-292 [125] and BLU-667 [126] were reported to demonstrate robust efficacy in the treatment of *RET*-fusion (and mutation) positive thyroid cancers. The overall response rate in 31 thyroid cancers (29 MTC, two PTC) treated with BLU-667 was 40%, while LOXO-292 had a 45% objective response rate in *RET*-mutant MTC and a 100% objective response rate in *RET*-rearranged PTC [116].

Studies evaluating BRAF inhibitor therapy (vemurafenib/dabrafenib) with and without MEK inhibition (trametinib) are ongoing in a multitude of adult and pediatric malignancies. Adult patients with RAI-refractory metastatic or unresectable BRAFV600E-positive PTCs treated with vemurafenib alone had a response rate of 38.5%, while patients with metastatic or advanced BRAFV600E-positive anaplastic thyroid carcinoma receiving combination dabrafenib and trametinib had a response rate of 69% [127,128]. To the best of our knowledge, use of these inhibitors for the treatment of pediatric thyroid cancer has not been reported. 

Most recently, multiple simultaneous studies evaluated the efficacy of TRK inhibitors in advanced solid tumors across three clinical trials comprised of a phase I trial in adults, a phase I/II trial in children (SCOUT), and a phase II basket trial in adults and adolescents (NAVIGATE) [129]. These trials contained a total of seven patients with *NTRK*-rearranged thyroid cancer. Of the patients with measurable disease (five adults), all demonstrated at least partial response (one responded completely) [129,130,131,132]. The two pediatric patients included in this cohort did not have measurable disease at the time of the trial; they remained stable on therapy at 16 months [130,131,132]. An additional case of pediatric thyroid cancer with TRK-inhibitor induced stabilization of disease was reported by Mahajan et al. [124]. Studies evaluating the efficacy of ALK inhibitors for tumors outside of *ALK*-rearranged non-small cell lung cancer (including thyroid cancer) are also underway [133], with a case report in the adult population demonstrating an excellent response to crizotinib (>90% reduction of pulmonary disease) [134].

## 7. Conclusions

In summary, pediatric thyroid carcinoma is a rare tumor but it is the most common carcinoma in children. The increased incidence of gene fusions in pediatric versus adult thyroid cancer is important to recognize as it has both potential implications for diagnostic molecular test selection (e.g., indeterminate FNA diagnoses) and offers novel therapeutic options. More data is needed to determine when alternative therapeutic strategies should be pursued, but targeted therapies have been used with great success in refractory pediatric thyroid cancer.

## Figures and Tables

**Table 1:** Molecular alterations in “pediatric” thyroid cancer by study.

	Age	Radiation Exposure	Diagnosis	*RET*-Fusion	*RET*-Mutation	*RAS*-Mutation	*BRAF*-Mutation	*BRAF*-Fusion	*NTRK*-Fusion	*ALK*-Fusion	*PPARG*-Fusion	Additional Targets	Notes
Fugazzola L et al. [55]	6–14 years	Post-radiation	6 PTC	4/6 (67%)	NE	NE	NE	NE	0/6 (0%)	NE	NE		
Klugbauer et al. [56]	<11 years	Post-radiation	12 PTC	8/12 (67%)	NE	NE	NE	NE	NE	NE	NE		
Nikiforov et al. [57]	5–19 years	Post-radiation	33 PTC	NE	NE	0/33 (0%)	NE	NE	NE	NE	NE	*TP53*	
			1 FTC	NE	NE	1/1 (100%)	NE	NE	NE	NE	NE		
Bongarzone et al. [58]	4–19 years	Sporadic *	9 PTC	6/9 (67%)	NE	NE	NE	NE	1/9 (11%)	NE	NE		* Fusion-+ cases
Williams et al. [59]	7–14 years	Sporadic	21 PTC	**10/21 (48%)**	NE	NE	NE	NE	NE	NE	NE		
Nikiforov et al. [60]	5–18 years	Post-radiation	38 PTC	33/38 (87%)	NE	NE	NE	NE	NE	NE	NE		
		Sporadic	17 PTC	12/17 (71%)	NE	NE	NE	NE	NE	NE	NE		
Motomura et al. [61]	9–14 years	Sporadic	10 PTC	**3/10 (30%)**	NE	NE	NE	NE	NE	NE	NE		
Suchy et al. [62]	“children”	Post-radiation	34 PTC	NE	NE	0/34 (0%)	NE	NE	NE	NE	NE	*TP53*	
Pisarchik et al. [63] Pisarchik et al. [64]	<29 years	Post-radiation	32 PTC combined	10/32 (31%)	NE	NE	NE	NE	NE	NE	NE		
Fenton et al. [65]	6–21 years	Post-radiation	1 PTC	NE	NE	0/1 (0%)	NE	NE	NE	NE	NE		
		Sporadic	30 PTC	NE	NE	**2/30 (7%)**	NE	NE	NE	NE	NE		
			2 MTC	NE	NE	0/2 (0%)	NE	NE	NE	NE	NE		
			4 FTC	NE	NE	0/4 (0%)	NE	NE	NE	NE	NE		
Thomas et al. [66]	6–18 years	Post-radiation	67 PTC	37/67 (55%)	NE	NE	NE	NE	NE	NE	NE		
			7 FTC	0/7 (0%)	NE	NE	NE	NE	NE	NE	NE		
Beimfohr et al. [67]	0–19 years *	Post-radiation	81 PTC	RET Negative	NE	NE	NE	NE	6/81 (7%)	NE	NE		* at exposure
Fenton et al. [68]	6–21 years	Post-radiation	1 PTC	1/1 (100%)	NE	NE	NE	NE	NE	NE	NE		
		Sporadic	33 PTC	**15/33 (45%)**	NE	NE	NE	NE	NE	NE	NE		
			1 MTC	0/1 (0%)	NE	NE	NE	NE	NE	NE	NE		
			8 FTC	0/8 (0%)	NE	NE	NE	NE	NE	NE	NE		
Santoro et al. [69]	<15 years	Post-radiation	106 PTC	36/106 (34%)	NE	0/23–31 (0%)	NE	NE	NE	NE	NE	*TSHR*, *TP53*	
Rabes et al. [70]	≤14 years	Post-radiation	99 PTC	50/99 (50%)	NE	NE	NE	NE	4/99 (4%)	NE	NE		
	>14 years		92 PTC	44/92 (48%)	NE	NE	NE	NE	2/92 (2%)	NE	NE		
Pauws et al. [71]	9–16 years	Sporadic	8 PTC	NE	NE	**0/8 (0%)**	NE	NE	NE	NE	NE		
Elisei et al. [72]	<18 years	Post-radiation	25 PTC	19/25 (76%)	NE	NE	NE	NE	NE	NE	NE		
		Sporadic	25 PTC	**10/25 (40%)**	NE	NE	NE	NE	NE	NE	NE		
Nikifurova et al. [73]	6–31years	Post-radiation	55 PTC	32/55 (58%)	NE	NE	2/55 (4%)	NE	NE	NE	NE		
	9–77 years	Sporadic	82 PTC	16/82 (20%)	NE	NE	30/82 (37%)	NE	NE	NE	NE		
Kumagai et al. [74]	≤15 years	Post-radiation	48 PTC	5/15 (33%)	NE	0/14 (0%)	0/15 (0%)	NE	NE	NE	NE		
	>15 years			12/33 (36%)	NE	2/32–33 (6%)	8/33 (24%)	NE	NE	NE	NE		
	<15 years	Sporadic	29 PTC	NE	NE	**0/16–22 (0%)**	**0/30 (0%)**	NE	NE	NE	NE		
			1 FTC										
			1 PDTC				1/1 (100%)						
Lima et al. [75]	13–30 years	Post-radiation	34 PTC	14/34 (41%)	NE	NE	4/34 (12%)	NE	NE	NE	NE		
	<18 years	Sporadic	17 PTC	NE	NE	NE	**1/17 (6%)**	NE	NE	NE	NE		
Penko et al. [76]	10–21 years	Post-radiation	1 PTC	1/1 (100%)	NE	0/1 (0%)	0/1 (0%)	NE	NE	NE	NE		
		Sporadic/Unknown	13 PTC	**6/11 (55%)**	NE	**0/10 (0%)**	**0/13 (0%)**	NE	NE	NE	NE		
			1 MTC	0/1 (0%)	NE	0/1 (0%)	0/1 (0%)	NE	NE	NE	NE		
			4 FTC	0/3 (0%)	NE	0/2 (0%)	0/4 (0%)	NE	NE	NE	NE		
Rosenbaum et al. [77]	10–17 years	Sporadic	20 PTC *	NE	NE	NE	**4/20 (20%)**	NE	NE	NE	NE		* Select histology
Espadinha et al. [78]	5–21 years	Unknown	15 PTC	NE	NE	NE	**1/15 (7%)**	NE	NE	NE	NE		
Monaco et al. [54]	≤21 years	Unknown	X PTC *	3/X	NE	4/X	2/X	NE	NE	NE	2/X		* 66 FNA samples
			X FTC *	0/X	NE	0/X	0/X	NE	NE	NE	0/X		
Sassolas et al. [79]	8–19 years	Post radiation	5 PTC	8/28 (29%) *	NE	1/28 (4%) *	**2/28 (7%)**	NE	**0/28 (0%)**	NE	NE		* One case w/ two alterations
		Sporadic	23 PTC										
Ricarte-Filho et al. [80]	<23 years	Post-radiation	26 PTC	15/26 (58%)	0/26 (0%)	0/26 (0%)	3/26 (12%)	2/26 (8%)	3/26 (12%)	NE	2/26 (8%)	*TSHR*, *PIK3CA*, *AKT1*	one *TSHR*, two *TERT*
		Sporadic	27 PTC	**7/27 (26%)**	**0/27 (0%)**	**2/27 (7%)**	**7/27 (26%)**	**0/27 (0%)**	**2/27 (7%)**	NE	**0/27 (0%)**		
Leeman et al. [81] Leeman et al. [82]	14–35 years	Post-radiation	62 PTC	22/62 (35%) *	NE	6/62 (10%) *	9/62 (15%) *	NE	9/62 (15%) *	NE	2/62 (3%) *	RNA-seq (subset)	Four tumors w/ two alterations
Henke et al. [83]	<22 years	Sporadic	27 PTC	NE	NE	NE	**17/27 (63%)**	NE	NE	NE	NE		
Givens et al. [84]	0–18 years	Unknown	19 PTC	NE	NE	NE	**7/19 (37%)**	NE	NE	NE	NE		
Mitusake et al. [85]	9–22 years	Post-radiation *	67 PTC **	7/67 (10%)	NE	0/67 (0%)	43/67 (64%)	0/67 (0%)	4/67 (6%)	NE	NE	*TERT*	* includes cases dx shortly after exposure ** four w/ possible *APC*-FAP
			1 PDTC	0/1 (0%)	NE	0/1 (0%)	0/1 (0%)	0/1 (0%)	0/1 (0%)	NE	NE		
Ballester et al. [86]	10–18 years	Post-radiation	2 PTC	1/2 (50%)	0/1 (0%)	0/1 (0%)	0/2 (%)	NE	0/1 (0%)	NE	0/1 (0%)	Ion Torrent AmpliSeq Cancer Hotspot Panel v.2	
		Sporadic	23 PTC	**5/23 (22%)**	**0/10 (0%)**	**0/10 (0%)**	**10/23 (43%)**	NE	**0/8 (0%)**	NE	**0/8 (0%)**		
			1 MTC	0/1 (0%)	1/1 (100%)	0/1 (0%)	0/1 (0%)	NE	NE	NE	NE		
			1 FTC	0/1 (0%)	0/1 (0%)	0/1 (0%)	0/1 (0%)	NE	NE	NE	NE		One *CTNNB1*
Picarsic et al. [87]	<18 years	Sporadic	17 PTC	**3/17 (18%)**	**0/17 (0%)**	**3/17 (18%)**	**3/17 (18%)**	0/17 (0%) *	4/17 (24%) *	0/17 (0%) *	**1/17 (6%)**	Thyroseq2 (subset)	* combined denominator
			1 FTC	0/1 (0%)	0/1 (0%)	0/1 (0%)	0/1 (0%)	NE	NE	NE	0/1 (0%)		
Prasad et al. [88]	6–18 years	Sporadic	27 PTC	**6/27 (22%)**	**0/27 (0%)**	**0/27 (0%)**	**13/27 (48%)**	**0/27 (0%)**	**7/27 (26%)**	**0/27 (0%)**	**0/27 (0%)**	Thyroseq2	
Nikita et al. [89]	7–18 years	Post-radiation	2 PTC	0/2 (0%)	NE	0/2 (0%)	0/2 (0%)	NE	NE	NE	0/2 (0%)	*TERT*	
		Sporadic	32 PTC	**6/32 (19%)**	NE	**1/32 (3%)**	**9/32 (28%)**	NE	NE	NE	**3/32 (9%)**		
			5 FTC	0/5 (0%)	NE	1/5 (20%)	0/5 (0%)	NE	NE	NE	1/5 (20%)		
Alzahrani et al. [90]	9–18 years	Unknown	52 PTC	NE	NE	NE	**12/52 (23%)**	NE	NE	NE	NE	*TERT*	One *TERT*
			2 FTC	NE	NE	NE	NE	NE	NE	NE	NE		
			1 PDTC	NE	NE	NE	0/1 (0%)	NE	NE	NE	NE		
Gertz et al. [91]	8–18 years	Sporadic	14 PTC	2/14 (14%) *	NE	**0/14 (0%)**	5/13 (38%) *	NE	NE	NE	NE		One tumor w/ both
Cordioli et al. [92]	4–18 years	Sporadic	30 PTC	NE	NE	NE	NE	**3/30 (10%)**	NE	NE	NE		
Onder et al. [45]	≤18 years	Post-radiation	3 PTC	NE	NE	NE	**15/50 (30%)**	NE	NE	NE	NE	*TERT*	
		Sporadic	47 PTC										
Cordioli et al. [93]	≤18 years	Post-radiation	3 PTC	13/35 (37%) *	NE	**0/35 (0%)**	**3/35 (9%)**	4/35 (11%) *	3/35 (9%) *	NE	NE		* Three cases w/ two alterations
		Sporadic	32 PTC										
Alzahrani et al. [94]	≤18 years	Unknown	72 cPTC 7 FVPTC	NE	NE	**2/79 (3%)**	**19/79 (24%)**	NE	NE	NE	NE	*PTEN*, *PIK3CA*, *TERT*	one *PTEN*, two *PIK3CA*, one *TERT*
Vanden Borre et al. [95]	≤21 years	Unknown	14 PTC	**5/14 (36%)**	0/14 (0%)	**0/14 (0%)**	**2/14 (14%)**	**0/14 (0%)**	**0/14 (0%)**	**3/14 (21%)**	NE	236 cancer genes and 14/19 fusion genes	
			2 MTC	0/2 (0%)	2/2 (100%)	0/2 (0%)	0/2 (0%)	0/2 (0%)	0/2 (0%)	0/2 (0%)	NE		
Hardee et al. [96]	≤21 years	Unknown	50 PTC	NE	NE	NE	**24/50 (48%)**	NE	NE	NE	NE		
Oishi et al. [97]	≤20 years	Sporadic	81 PTC	NE	NE	NE	**44/81 (54%)**	NE	NE	NE	NE	*CTNNB1*, *PIK3CA*, *TERT*	
Vuong et al. [98]	<21 years	Sporadic	41 FTC	NE	NE	5/41 (12%)	NE	NE	NE	NE	0/39 (0%)		
Mostoufi-Moab et al. [99]	≤18 years	Post-radiation	3 PTC	0/3 (0%)	NE	1/3 (33%)	0/3 (0%)	NE	NE	NE	0/3 (0%)		
		Sporadic	59 PTC	**12/59 (20%)**	NE	**3/59 (5%)**	**12/59 (20%)**	NE	NE	NE	**2/59 (3%)**		
			6 FTC	0/6 (0%)	NE	2/6 (33%)	0/6 (0%)	NE	NE	NE	0/6 (0%)		
Wasserman et al. [100]	<18 years	Post-radiation	2 PTC	0/2 (0%)	NE	0/2 (0%)	0/2 (0%)	NE	NE	NE	0/2 (0%)	*DICER1*, *TERT*	Three pts w/ *DICER1* mutations
		Sporadic	28 PTC	**7/28 (25%)**	NE	**0/28 (0%)**	**5/28 (18%)**	NE	NE	NE	**0/28 (0%)**		
Huang et al. [101]	<21 years		59 PTC	NE	NE	NE	16/30 (53%)	NE	NE	NE	NE		
		Unknown	5 FTC										
			1 MTC										
Sisdelli et al. [102]	≤18 years	Post-radiation	3 PTC	NE	NE	NE	0/3 (0%)	1/3 (33%)	NE	NE	NE		
		Sporadic	77 PTC	NE	NE	NE	**12/77 (16%)**	**14/77 (18%)**	NE	NE	NE		
Pekova et al. [103]	6–20 years	Post-radiation	2 PTC	0/2 (0%)	NE	**2/83 (2%)**	**15/83 (18%)**	NE	NE	NE	NE	*IDH1*, *CHEK2*, *PPMID*, *TERT*, *EIF1AX*, *EZH1*	One Cowden’s patient
		Sporadic	81 PTC	**18/81 (22%)**									

Bolded results were used in the calculation of overall percentage of sporadic PTC harboring these alterations; *, ** asterisks correspond to information regarding the study within that row. cPTC = classic PTC, dx= diagnosed, FA = follicular adenoma, FTC = follicular thyroid carcinoma, FVPTC = follicular variant of PTC, IHC = immunohistochemistry, NE = not evaluated, PDTC = poorly differentiated thyroid carcinoma, PTC = papillary thyroid carcinoma, MTC = medullary thyroid carcinoma, w/ = with, X = unknown.

**Table 2:** Therapies available by molecular alteration and/or histology.

Histology	Molecular Alteration	Targeted Therapy (Clinicaltrials.gov ID)
MTC	Presumed *RET* mutation	vandetanib cabozantinib
Thyroid Cancer (MTC, PTC)	*RET* mutations and fusions	BLU-667 (NCT03037385) LOXO-292 (NCT03157128; NCT03899792)
DTC	Non-specific	vandetanib (NCT01876784) cabozantinib (NCT03690388) sorafenib lenvatinib
PTC/ATC	BRAFV600E	vemurafenib dabrafenib/trametinib
Solid tumors (including thyroid)	*NTRK*-fusion	larotrectinib
PTC	*ALK*-fusion	crizotinib (NCT02034981) ceritinib (NCT02289144) alectinib (NCT03194893)
